# Global surge in pancreatic cancer cases driven by ageing populations and modifiable risks

**DOI:** 10.7189/jogh.16.04032

**Published:** 2026-01-30

**Authors:** Wanyi Zheng, Guojia Jiang, Jolaoluwa Grace Oparinde, Ziqi Zhang, Deji Song, Yi Ding, Jiayun Feng, Youyan Xu, Danni Xu, Hailei Zhao, Li Zhang, Guang Ji, Lili Lu

**Affiliations:** 1 Shanghai University of Traditional Chinese Medicine, School of Public Health, Shanghai, China; 2 Longhua Hospital, Shanghai University of Traditional Chinese Medicine, Institute of Digestive Diseases, Shanghai, China; 3 State Key Laboratory of Integration and Innovation of Classical Formula and Modern Chinese Medicine, Shanghai, China

## Abstract

**Background:**

Pancreatic cancer (PC) ranks among the most lethal malignant neoplasms, primarily due to its late-stage diagnosis and lack of available therapeutic modalities. This study aimed to characterise the current PC epidemiological profiles, lifestyle-related contributors, and projections to unveil its global disease burden.

**Methods:**

Using 2022 data from the Global Cancer Observatory (GCO), we estimated PC incidence, mortality, 5-year prevalence, and mortality-incidence-ratio. Modifiable risk factors were extracted from the Global Health Observatory to identify its predictive model. The temporal trends were assessed via estimated annual percentage changes (EAPCs) stratified by age and gender, and the future projection was also collected from GCO, 2022–2050, estimated number of new cases and deaths data.

**Results:**

In 2022, Northern America and Europe had the highest PC burden, with males consistently affected more than females. Alarmingly, a concerning increase in PC mortality was observed among older females. Projections indicate an 85–91% increase in elderly PC cases by 2050, with Asia facing the greatest challenge (487 087 estimated new cases) and Africa being estimated with the fastest mortality growth (159.2%). Strong positive correlations were observed between PC prevalence and human development index (HDI), as well as lifestyle factors *e.g*. raised total cholesterol (correlation coefficient (*r*) = 0.695, *P* < 0.001), cigarette smoking (*r* = 0.528, *P* < 0.001), and alcohol drinking (*r* = 0.505, *P* < 0.001).

**Conclusions:**

This research underlines the urgent need for region-specific interventions, not only for Northern America and Europe, which currently bear a high PC burden, but also for high-risk populations like Asia and Africa. The projected 85–91% rise in elderly PC cases by 2050, coupled with emerging risks in young females (incidence rose in n/N = 39/52 countries), demands prioritised research on modifiable factors and sex-tailored prevention strategies. These discoveries call for global action to mitigate the escalating PC burden through demographic-targeted public health measures.

Pancreatic cancer (PC) is among the most lethal malignant neoplasms with a reported ever-rising incidence and prevalence, as well as an overall 5-year survival rate of merely 12% [1]. Lack of specific symptoms at the preliminary stage and feasible population-based screening programmes contribute to its late diagnosis and poor prognosis [2]. Accumulating evidence suggests strong associations between lifestyle-related risk factors (*e.g*. obesity, type 2 diabetes mellitus, smoking) and PC [3–10] as these factors contribute to pancreatic diseases like acute and chronic pancreatitis that predispose to PC. Additionally, PC incidence, mortality, and 5-year survival are also reported to correlate with age and gender [11,12].

Although existing researches [13–15] have unveiled unilateral epidemiological profiles and lifestyle-related risk assessments of PC at individual, regional, or national levels, these studies have not performed stratified analyses covered all age groups or different global regions, across the globe. Furthermore, these investigations often lack a detailed and multi-dimensional way. Therefore, a more wide-ranging and elaborate data analysis is required for PC.

Utilising data from the World Health Organization (WHO) Global Cancer Observatory (GCO) and the Global Health Observatory (GHO), this study comprehensively characterises current global PC epidemiological profiles, lifestyle-related contributors, and future projections. Our study advances existing knowledge in three key aspects: first, we conducted a stratified analysis of PC incidence, mortality, and mortality-to-incidence ratio (MIR) by human development index (HDI) tiers, addressing the lack of global multi-level regional analysis. Second, we examined country-level associations between HDI, risk factors, and 5-year prevalence to identify key drivers of PC, filling the gap in integration between socioeconomic status, lifestyle, and PC burden. Third, we analysed age- and gender-specific estimated annual percentage changes (EAPCs) in incidence and mortality, and projected future PC burden from 2022–2050, overcoming the lack of comprehensive age-gender stratification and long-term projection in prior research. This approach lays a more robust groundwork for investigating novel therapeutic approaches and targeted interventions across divergent socioeconomic contexts.

## METHODS

### Data collection

This study followed Strengthening the Reporting of Observational studies in Epidemiology (STROBE) reporting guidelines (File S1 in the **Online Supplementary Document**).

We sourced PC incidence, mortality, and 5-year prevalence data for 2022 from the WHO GCO 'Cancer Today' data visualisation tool [16]. Age-standardised prevalence data for risk factors including obesity (2022), hypertension (2019), diabetes (2022), raised total cholesterol (2008), cigarette smoking (2022), alcohol drinking (2020), and insufficient physical activity (2022), were extracted from the GHO database of WHO [17]. We used the GCO 'Cancer Over Time' visualisation tools to collect EAPC data [18]. The national HDI values for 2022 were acquired from the United Nations Development Programme (UNDP) [19].

### Study population

PC (C13) cases were identified according to ICD-10 codes [20]. Our analysis encompassed lifestyle-associated risk factor data: obesity (199 countries), hypertension (195 countries), diabetes (199 countries), raised total cholesterol (189 countries), cigarette smoking (165 countries), alcohol drinking (187 countries), and insufficient physical activity (195 countries); incidence, mortality, and 5-year prevalence data from 185 countries, and HDI data from 192 countries for association analyses. EAPC data spanned 52 countries/regions for incidence and 68 for mortality. All epidemiological metrics were analysed using existing WHO GCO data sets. The association analyses using both of WHO GCO and GHO databases was restricted to countries with available data in both sources.

### Outcomes and variables

Study outcomes comprised globally assessed age-standardised incidence, mortality, and 5-year prevalence of PC. Key variables included: age (< 50 years; ≥ 50 years); sex (male and female) [1,21,22]; and HDI categorised per UNDP standards as very high (≥ 0.8), high (0.7–0.799), medium (0.55–0.699), and low (< 0.55). The cutoffs were selected based on the Human Development Report 2022 [19]. Lifestyle-associated risk factors were operationally defined as: obesity (body mass index (BMI) ≥ 30 kg/m^2^); hypertension (systolic blood pressure ≥ 140 mmHg or diastolic blood pressure ≥ 90 mmHg); diabetes (fasting glucose ≥ 7.0 mmol/L, clinical history, or glucose-lowering medication use); raised total cholesterol (≥ 5.0 mmol/L); cigarette smoking (any tobacco use, daily or occasional); alcohol drinking (annual pure alcohol volume per capita); and insufficient physical activity (≤ 150-minute/week moderate-intensity exercise).

### Statistical analysis

The PC incidence and mortality rates were rendered as age-standardised rates (ASR) per 100 000 persons [23]. The 5-year prevalence represents the specific number of individuals who have been diagnosed with PC including both newly diagnosed cases and existing cases from prior periods, given as a proportion of the population per 100 000 individuals. The magnitude of temporal trend changes in cancer incidence and mortality was characterised using EAPCs, which were calculated by fitting a simple regression model to the log of the ASRs [24]. The correlations between HDI in 2022, lifestyle-related risk factors, and the 5-year prevalence of PC were evaluated using Spearman’s correlation analysis and were presented by the correlation coefficient (*r*) and 95% confidence intervals (95% CIs). All countries/regions were divided into two groups (high and low-prevalence) in light of the median value of the 5-year prevalence of PC, to identify the potential risk factors. The associations between risk factors and PC prevalence were evaluated on a country level using the logistic regression analysis, and the data were presented as odds ratios (ORs) and 95% CIs. Further, to predict the risk of PC using lifestyle-associated factors, we calculated the prediction probabilities of investigated variables through logistic regression models and analysed trends using receiver operating characteristic (ROC) curves. The area under the curves (AUCs) and 95% CIs were used to assess model accuracy.

Statistical analyses were performed using SPSS, version 27.0 (IBM Corp., Armonk, NY, USA), with a statistical significance threshold of *P* ≤ 0.05.

## RESULTS

### Global Incidence and Mortality of PC in 2022

**Figure 1:**
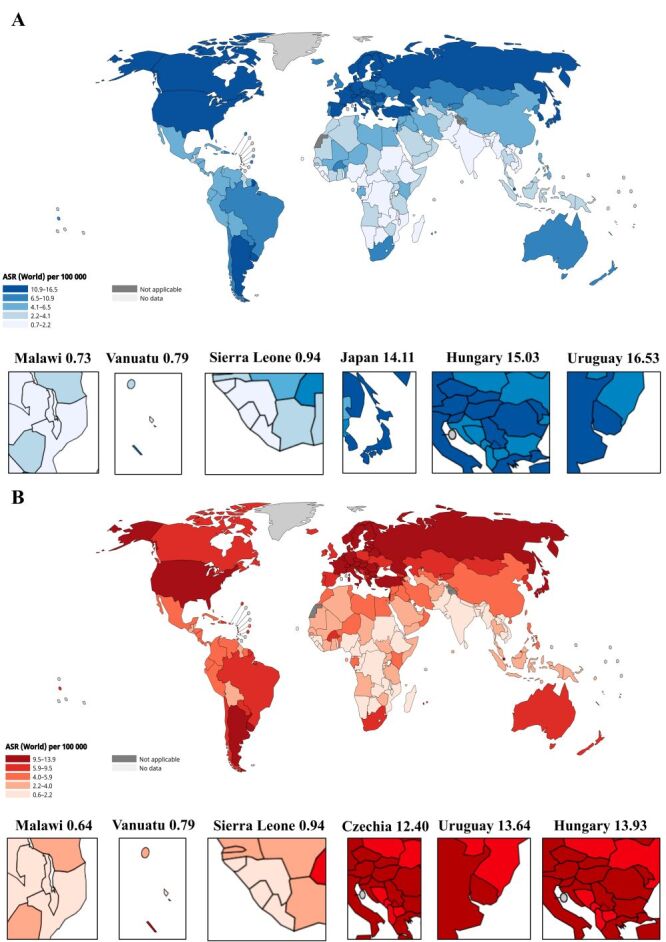
Global epidemiological profiles of PC in 2022. **Panel A.** The ASR of PC incidence, per 100 000, including the top and lowest three countries. **Panel B.** The ASR of PC mortality, per 100 000, including the top and lowest three countries [16]. Free to use under IARC Publications Terms of Use and Conditions. ASR – age-standardised rate, PC – pancreatic cancer.

In 2022, the global age-standardised incidence rate of PC averaged 6.22/100 000. Uruguay had the highest rate of 16.53/100 000, followed by Hungary (15.03/100 000) and Japan (14.11/100 000). The lowest incidence rates were seen in Malawi (0.73/100 000), Vanuatu (0.79/100 000), and Sierra Leone (0.94/100 000) (**Figure 1**, panel A, **Table 1**). The global mortality for PC was 5.74 per 100 000, with the highest mortality in Hungary (13.93/100 000), followed by Uruguay (13.64/100 000) and Czechia (12.40/100 000), while the lowest rate was in Malawi (0.64/100 000), Vanuatu (0.79/100 000) and Sierra Leone (0.94/100 000) (**Figure 1**, panel B, **Table 1**).

**Table 1:** ASRs of incidence, mortality and 5-year prevalence of pancreatic cancer

Countries/regions	Both sexes			Males			Females		
	**Incidence**	**Mortality**	**Prevalence**	**Incidence**	**Mortality**	**Prevalence**	**Incidence**	**Mortality**	**Prevalence**
**Afghanistan**	2.76	2.61	2.16	3.25	2.11	2.40	2.20	2.30	1.94
**Albania**	7.60	7.41	5.28	10.3	7.01	7.01	4.85	5.10	3.67
**Algeria**	3.71	3.60	2.67	4.45	2.99	3.03	2.89	2.98	2.31
**Angola**	2.31	2.25	2.22	2.99	1.97	3.10	1.66	1.66	1.38
**Argentina**	11.45	10.39	7.93	13.48	8.45	9.04	8.88	9.78	6.99
**Armenia**	13.40	12.16	9.45	18.85	11.58	13.48	8.85	9.45	6.41
**Australia**	10.08	8.49	6.96	10.42	6.73	6.84	7.30	9.73	7.08
**Austria**	13.54	11.67	9.51	15.34	9.37	10.23	9.97	11.92	8.87
**Azerbaijan**	4.33	3.84	3.35	5.39	3.34	3.88	3.08	3.50	2.92
**Bahamas**	4.54	4.23	2.96	6.10	3.77	4.06	3.31	3.31	2.04
**Bahrain**	4.06	3.98	2.47	3.83	2.56	2.05	4.41	4.41	3.12
**Bangladesh**	1.28	1.23	1.10	1.46	0.96	1.25	1.05	1.08	0.93
**Barbados**	8.85	8.55	5.84	11.42	7.43	7.33	6.74	6.74	4.57
**Belarus**	10.49	9.59	7.83	15.77	10.01	11.22	6.11	6.87	5.34
**Belgium**	10.90	10.24	7.53	12.39	8.04	7.93	8.97	9.59	7.21
**Belize**	3.69	3.29	2.16	2.53	1.17	1.86	4.86	4.86	2.49
**Benin**	4.56	4.47	3.15	5.55	3.63	3.75	3.75	3.69	2.63
**Bhutan**	2.87	2.87	1.94	4.16	2.87	2.79	1.39	1.39	0.96
**Bolivia (Plurinational State of)**	4.28	3.88	2.76	4.49	2.86	2.76	3.60	4.05	2.78
**Bosnia Herzegovina**	10.27	9.31	7.93	12.08	7.77	8.89	7.59	8.67	7.09
**Botswana**	1.09	1.09	0.89	1.32	0.91	0.98	0.92	0.92	0.83
**Brazil**	6.84	6.63	4.80	8.01	5.36	5.41	5.67	5.85	4.27
**Brunei Darussalam**	5.77	5.96	3.69	5.11	3.99	3.06	6.25	6.42	4.33
**Bulgaria**	10.86	10.40	7.89	13.76	9.21	9.44	7.90	8.51	6.60
**Burkina Faso**	9.52	8.84	8.81	7.30	4.57	5.65	10.98	11.67	11.69
**Burundi**	2.17	2.17	1.66	2.20	1.52	1.65	2.14	2.14	1.65
**Cambodia**	1.99	1.86	1.71	2.57	1.70	1.96	1.43	1.58	1.53
**Cameroon**	2.00	1.86	1.55	3.08	1.96	2.33	1.01	1.04	0.84
**Canada**	10.89	9.20	7.57	12.05	7.25	7.97	7.97	9.81	7.20
**Cape Verde**	1.77	2.04	1.21	3.79	1.93	2.36	1.33	0.72	0.50
**Central African Republic**	1.80	1.80	1.37	2.51	1.73	1.72	1.31	1.31	1.12
**Chad**	2.96	2.78	2.66	2.87	1.94	2.16	2.82	3.10	3.16
**Chile**	7.96	7.45	5.40	8.35	5.40	5.41	7.12	7.61	5.40
**China**	6.43	5.62	4.59	7.67	4.73	5.28	4.43	5.25	3.92
**Colombia**	5.78	5.26	4.02	6.25	3.92	4.27	4.89	5.37	3.80
**Comoros**	1.10	1.10	0.92	1.12	0.77	1.10	1.07	1.07	0.74
**Congo, Democratic Republic of**	2.08	2.03	1.58	2.27	1.53	1.62	1.90	1.93	1.56
**Congo, Republic of**	1.63	1.63	1.37	1.86	1.28	1.44	1.46	1.46	1.32
**Costa Rica**	6.06	5.85	3.88	6.50	4.17	4.25	5.66	5.59	3.52
**Croatia**	10.55	10.18	7.08	12.55	8.46	8.10	8.41	8.91	6.20
**Cuba**	6.34	5.64	4.60	7.30	4.48	5.10	4.83	5.43	4.14
**Cyprus**	12.42	11.88	8.16	14.53	9.89	9.85	9.49	10.29	6.54
**Czechia**	13.53	12.40	9.44	15.12	9.86	9.98	10.65	12.14	9.00
**Côte d'Ivoire**	3.36	3.30	2.54	3.26	2.22	2.46	3.37	3.46	2.62
**Denmark**	12.31	11.40	8.06	13.1	8.66	8.33	10.27	11.51	7.78
**Djibouti**	1.97	1.97	1.46	2.22	1.53	1.29	1.80	1.80	1.69
**Dominican Republic**	7.26	6.43	5.68	8.36	5.13	6.26	5.50	6.24	5.14
**Ecuador**	5.28	4.70	3.63	4.92	3.01	3.29	4.97	5.56	3.94
**Egypt**	5.54	5.29	4.36	7.15	4.70	5.36	3.94	4.10	3.44
**El Salvador**	3.79	3.50	2.70	4.68	2.97	3.18	2.92	3.16	2.36
**Equatorial Guinea**	3.41	3.41	3.05	3.96	2.74	3.53	2.67	2.67	2.42
**Eritrea**	1.76	1.72	1.25	1.98	1.37	1.25	1.53	1.61	1.28
**Estonia**	12.44	11.55	8.14	14.39	9.26	9.01	9.99	11.08	7.53
**Eswatini**	1.97	1.97	1.56	2.40	1.65	1.83	1.61	1.61	1.35
**Ethiopia**	2.00	1.81	1.68	2.15	1.35	1.61	1.70	1.88	1.75
**Fiji**	4.09	4.09	2.39	5.31	3.66	2.98	3.03	3.03	1.87
**Finland**	12.93	12.18	8.40	14.33	9.59	8.88	10.56	11.67	7.97
**France (metropolitan)**	13.61	11.57	9.74	16.12	9.39	11.23	9.74	11.27	8.37
**France, Guadeloupe**	11.87	11.20	8.27	13.28	8.80	8.84	9.96	10.70	7.73
**France, La Réunion**	8.69	7.74	6.11	11.62	7.45	7.90	5.08	6.16	4.51
**France, Martinique**	9.05	8.38	6.30	11.21	7.73	8.05	6.21	7.40	4.96
**French Guyana**	11.05	9.26	6.50	15.03	8.93	8.26	6.59	7.97	4.79
**French Polynesia**	6.62	5.85	4.05	10.82	6.41	7.07	2.45	2.45	0.98
**Gabon**	4.97	4.72	4.11	4.60	2.97	3.58	4.96	5.14	4.59
**Gaza Strip and West Bank**	6.69	6.56	4.37	8.58	5.91	5.52	4.79	5.01	3.30
**Georgia**	7.78	7.05	5.61	8.57	5.29	6.26	6.47	7.07	4.97
**Germany**	12.60	11.90	8.47	14.41	9.61	9.30	10.03	10.97	7.71
**Ghana**	2.47	2.40	2.08	2.65	1.77	1.95	2.30	2.35	2.24
**Greece**	11.45	10.25	8.03	14.07	8.70	9.70	8.15	9.10	6.49
**Guam**	6.16	5.62	3.19	8.44	5.03	4.28	4.23	4.23	2.10
**Guatemala**	3.62	3.33	2.61	4.11	2.62	2.81	2.96	3.23	2.44
**Guinea**	1.17	1.16	1.02	1.48	1.00	1.34	0.95	0.95	0.80
**Guinea-Bissau**	1.36	1.36	1.02	2.02	1.39	1.57	0.83	0.83	0.57
**Guyana**	2.96	2.68	2.08	3.51	1.97	2.56	2.43	2.43	1.60
**Haiti**	4.67	4.18	3.18	5.17	3.19	3.74	3.70	4.10	2.66
**Honduras**	5.35	4.84	3.97	6.41	3.60	4.27	4.65	4.51	3.75
**Hungary**	15.03	13.93	10.49	17.96	11.74	12.07	11.52	12.69	9.18
**Iceland**	10.49	8.80	6.94	12.33	6.91	8.45	7.52	8.75	5.41
**India**	1.39	1.31	1.12	1.79	1.16	1.35	0.94	1.01	0.88
**Indonesia**	2.88	2.95	2.00	3.38	2.51	2.23	2.39	2.48	1.81
**Iran, Islamic Republic of**	5.62	5.23	4.05	7.10	4.30	4.81	4.22	4.20	3.31
**Iraq**	4.68	4.63	3.48	6.72	4.58	4.78	2.94	2.97	2.35
**Ireland**	10.01	8.31	6.66	11.34	6.57	7.19	7.21	8.77	6.16
**Israel**	11.86	10.82	8.01	13.93	8.95	9.17	8.90	9.98	6.95
**Italy**	11.56	10.43	7.79	13.07	8.18	8.48	9.13	10.18	7.17
**Jamaica**	5.36	4.89	3.96	6.01	3.86	4.18	4.24	4.77	3.76
**Japan**	14.11	11.64	9.21	17.94	9.57	11.85	9.55	10.33	6.60
**Jordan**	5.57	5.46	4.51	6.89	4.61	5.37	4.27	4.27	3.64
**Kazakhstan**	7.61	7.51	5.78	9.27	6.38	6.80	6.21	6.33	4.98
**Kenya**	5.12	5.06	3.59	5.36	3.69	3.27	5.00	5.11	3.93
**Korea, Democratic People Republic of**	6.75	6.47	4.75	8.17	5.42	5.54	5.40	5.65	4.09
**Korea, Republic of**	10.89	8.59	7.97	11.86	7.26	8.00	6.88	9.98	8.02
**Kuwait**	4.73	4.67	2.93	4.92	3.37	3.00	4.43	4.48	2.91
**Kyrgyzstan**	6.77	6.29	4.89	7.81	5.07	5.53	5.45	5.92	4.34
**Lao People's Democratic Republic**	1.98	1.87	1.67	2.31	1.51	1.76	1.60	1.70	1.60
**Latvia**	13.44	12.21	9.19	17.42	11.09	11.57	9.58	10.73	7.34
**Lebanon**	5.67	5.60	4.23	6.76	4.62	4.81	4.50	4.56	3.60
**Lesotho**	1.79	1.79	1.16	2.84	1.96	1.98	0.99	0.99	0.52
**Liberia**	2.06	2.00	1.60	2.01	1.34	1.58	2.02	2.08	1.63
**Libya**	5.57	5.04	3.94	6.62	4.06	4.40	4.39	4.71	3.55
**Lithuania**	12.24	11.64	8.77	16.05	10.32	11.42	9.24	9.38	6.62
**Luxembourg**	9.46	9.09	6.12	12.39	8.37	7.00	6.26	7.14	5.38
**Madagascar**	1.82	1.81	1.48	2.09	1.42	1.77	1.56	1.56	1.19
**Malawi**	0.73	0.64	0.74	0.54	0.35	0.44	0.79	0.92	1.01
**Malaysia**	4.02	3.90	3.34	4.30	2.87	3.43	3.62	3.71	3.22
**Maldives**	2.78	2.78	1.60	4.34	2.99	2.33	1.05	1.05	0.72
**Mali**	4.13	3.80	3.32	5.24	3.30	3.97	2.97	3.20	2.77
**Malta**	13.01	12.03	9.03	15.08	9.58	10.56	10.13	10.74	7.42
**Mauritania**	2.56	2.56	1.86	3.52	2.43	2.44	1.74	1.74	1.35
**Mauritius**	5.56	4.22	4.81	5.39	3.28	3.82	3.75	5.89	5.88
**Mexico**	5.59	5.04	4.05	5.98	3.72	4.20	4.73	5.25	3.93
**Mongolia**	7.89	7.27	5.98	9.16	5.96	6.49	6.09	6.89	5.60
**Montenegro**	8.34	7.90	5.71	10.37	6.75	6.69	6.42	6.78	4.92
**Morocco**	4.12	3.95	3.15	5.10	3.39	3.74	3.07	3.21	2.60
**Mozambique**	1.27	1.21	1.00	1.76	1.12	1.41	0.89	0.89	0.67
**Myanmar**	1.94	1.91	1.50	2.47	1.67	1.78	1.54	1.56	1.30
**Namibia**	1.78	1.78	1.43	2.67	1.84	2.16	1.15	1.15	0.89
**Nepal**	1.99	1.90	1.53	1.81	1.20	1.39	2.05	2.15	1.66
**New Caledonia**	9.46	9.08	6.44	12.17	8.40	8.54	6.31	7.05	4.39
**New Zealand**	8.75	8.16	5.95	9.71	5.99	6.11	7.66	7.87	5.80
**Nicaragua**	5.55	5.10	3.88	6.19	3.95	4.05	4.64	5.09	3.79
**Niger**	4.88	4.65	3.47	7.94	5.31	5.09	1.85	2.08	1.94
**Nigeria**	1.95	1.89	1.39	2.24	1.50	1.53	1.63	1.68	1.27
**North Macedonia**	9.45	8.94	6.82	10.75	6.94	7.78	7.80	8.14	5.90
**Norway**	11.71	10.75	7.23	13.07	8.31	7.81	9.45	10.34	6.64
**Oman**	4.01	3.95	2.88	5.85	3.97	3.82	1.88	1.88	1.65
**Pakistan**	1.00	0.96	0.78	1.46	0.97	1.08	0.52	0.53	0.47
**Panama**	4.45	3.95	2.89	5.01	3.12	3.22	3.41	3.91	2.58
**Papua New Guinea**	2.40	2.44	1.64	3.36	2.37	2.36	1.50	1.50	0.95
**Paraguay**	7.00	6.36	4.77	7.79	4.87	5.20	5.66	6.18	4.33
**Peru**	5.91	5.30	4.03	5.48	3.39	3.54	5.65	6.30	4.51
**Philippines**	5.90	5.78	4.27	6.64	4.48	4.59	5.20	5.29	4.00
**Poland**	9.70	9.44	6.84	11.67	7.84	8.19	7.74	7.94	5.60
**Portugal**	9.82	9.29	6.49	12.69	8.21	8.43	7.08	7.39	4.80
**Puerto Rico**	7.65	6.85	5.46	9.99	6.43	6.89	4.92	5.82	4.34
**Qatar**	3.00	2.91	2.16	2.23	1.50	1.69	4.48	4.67	3.75
**Republic of Moldova**	11.77	11.38	9.00	15.24	10.16	11.85	8.71	8.89	6.49
**Romania**	11.48	10.63	8.43	14.92	9.64	10.81	7.78	8.51	6.26
**Russian Federation**	11.26	10.38	8.51	14.49	9.47	10.58	7.96	8.95	6.97
**Rwanda**	1.05	1.02	0.97	1.48	0.99	1.31	0.65	0.67	0.66
**Saint Lucia**	5.15	5.15	2.89	5.51	3.80	3.35	4.84	4.84	2.47
**Samoa**	8.81	8.81	6.63	7.45	5.14	5.14	10.08	10.08	8.12
**Sao Tome and Principe**	3.28	3.28	3.65	2.04	1.41	2.81	4.35	4.35	4.36
**Saudi Arabia**	2.90	2.91	2.17	3.39	2.37	2.41	2.18	2.20	1.79
**Senegal**	2.62	2.56	2.11	3.47	2.36	2.69	1.93	1.99	1.68
**Serbia**	10.97	10.03	7.90	12.72	8.12	8.87	8.54	9.43	7.02
**Sierra Leone**	0.94	0.94	1.95	0.21	0.14	2.50	1.57	1.57	1.45
**Singapore**	11.14	10.73	7.67	13.12	8.57	8.59	9.09	9.18	6.74
**Slovakia**	12.48	11.69	8.70	15.62	10.11	10.88	9.09	9.69	6.69
**Slovenia**	12.07	10.51	8.38	15.26	9.30	10.27	7.78	9.11	6.56
**Solomon Islands**	2.52	2.52	1.07	3.8	2.62	1.53	1.30	1.30	0.61
**Somalia**	2.86	2.78	2.13	2.85	1.94	1.95	2.78	2.90	2.31
**South Africa**	7.62	6.73	5.67	8.65	5.34	5.86	5.95	6.97	5.62
**South Sudan**	2.64	2.43	1.99	2.81	1.77	1.83	2.35	2.53	2.16
**Spain**	10.91	9.21	7.99	11.98	7.56	8.29	7.60	9.95	7.74
**Sri Lanka**	1.20	1.19	0.90	1.58	1.08	1.09	0.89	0.90	0.75
**Sudan**	1.56	1.42	1.03	1.87	1.21	1.04	1.15	1.31	1.03
**Suriname**	5.46	5.04	3.61	6.44	3.79	4.43	4.76	4.76	2.94
**Sweden**	13.45	10.82	9.45	14.89	7.92	10.64	10.15	11.73	8.21
**Switzerland**	12.33	10.36	8.45	12.95	7.98	8.35	9.21	11.74	8.56
**Syrian Arab Republic**	6.08	5.56	4.29	8.39	5.32	5.59	3.73	4.10	3.12
**Tajikistan**	1.86	1.81	1.37	2.01	1.41	1.38	1.65	1.76	1.38
**Tanzania, United Republic of**	2.58	2.53	1.87	3.63	2.42	2.43	1.84	1.83	1.42
**Thailand**	3.68	3.62	2.76	4.99	3.39	3.56	2.55	2.59	2.06
**The Netherlands**	11.74	10.33	8.38	12.51	7.73	8.43	9.51	11.03	8.33
**The Republic of the Gambia**	2.25	2.25	1.98	1.86	1.29	1.59	2.61	2.61	2.33
**Timor-Leste**	2.54	2.54	1.71	2.58	1.78	1.82	2.49	2.49	1.57
**Togo**	2.13	2.12	1.44	2.68	1.84	1.72	1.64	1.64	1.17
**Trinidad and Tobago**	6.75	6.14	4.34	8.79	5.44	5.52	4.65	4.97	3.27
**Tunisia**	4.73	4.61	3.52	6.19	4.15	4.57	3.34	3.40	2.55
**Turkmenistan**	3.73	3.55	2.83	5.03	3.30	3.59	2.65	2.78	2.29
**Türkiye**	11.13	10.70	7.86	14.92	10.07	10.51	7.52	7.86	5.55
**Uganda**	2.33	2.25	1.79	1.86	1.25	1.37	2.60	2.70	2.12
**Ukraine**	9.33	8.72	7.15	13.23	8.51	9.91	6.20	6.50	5.03
**United Arab Emirates**	3.45	3.35	2.00	3.48	2.31	1.95	3.61	3.64	2.30
**UK**	9.65	8.67	6.63	10.75	6.72	7.06	7.70	8.63	6.23
**USA**	12.40	9.64	9.06	14.17	7.76	9.73	8.20	10.83	8.47
**Uruguay**	16.53	13.64	11.21	18.83	10.69	12.41	12.20	14.57	10.17
**Uzbekistan**	5.34	4.74	4.36	6.22	3.83	4.82	4.06	4.60	3.97
**Vanuatu**	0.79	0.79	0.55	1.56	1.08	1.08	N/A	N/A	N/A
**Venezuela**	6.14	5.52	4.39	6.64	4.12	4.59	5.10	5.69	4.21
**Viet Nam**	1.47	1.45	1.19	2.10	1.43	1.59	0.94	0.97	0.84
**Yemen**	2.88	2.60	2.54	3.43	2.19	2.23	2.29	2.59	2.92
**Zambia**	2.03	1.87	1.64	2.56	1.68	1.90	1.50	1.67	1.47
**Zimbabwe**	3.91	3.79	2.66	4.97	3.31	3.25	3.19	3.28	2.29

ASR – age-standardised rate

**Figure 2:**
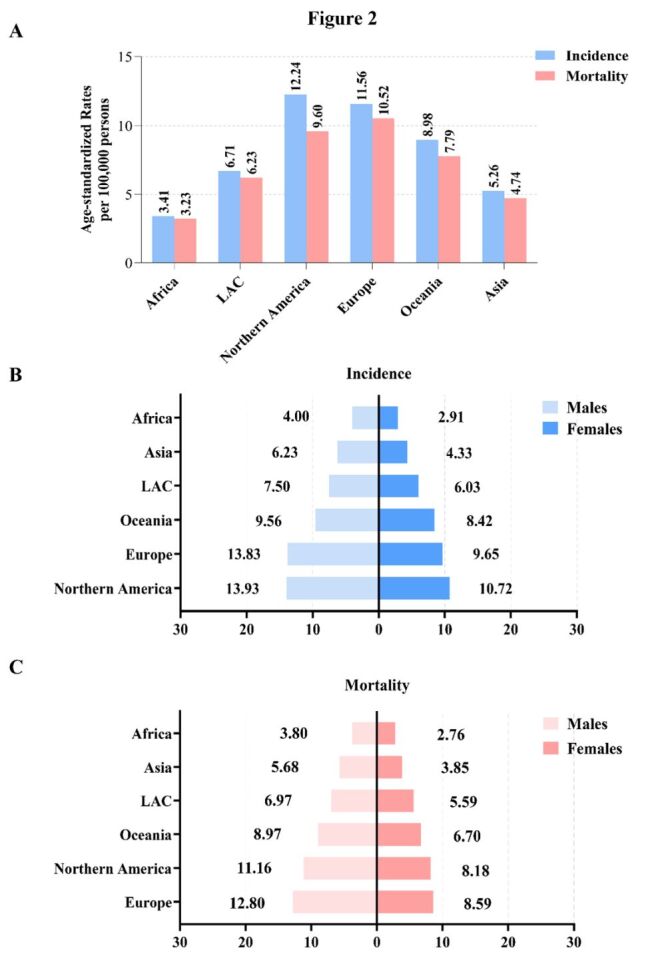
Geographical and sex disparities in the global burden of PC. **Panel A.** Bar plot depicting the global distribution of PC incidence and mortality across continents. **Panel B.** The ASR of PC incidence stratified by sex per 100 000. **Panel C.** The ASR of PC mortality stratified by gender, per 100 000 [16]. ASR – age-standardised rate, LAC – Latin America and the Caribbean, PC – pancreatic cancer.

When stratified by continent, the higher incidence and mortality were observed in Northern America (incidence = 12.24/100 000; mortality = 9.60/100 000) and Europe (incidence = 11.56/100 000; mortality = 10.52/100 000) (**Figure 2**, panel A). Further stratification by sex revealed consistently higher rates of both incidence and mortality among males. This gender disparity was particularly pronounced in Asia and Europe, where rates were approximately 1.5-fold higher in male compared to female (**Figure 2**, panels B–C).

### Trends in incidence stratified by age and gender

**Figure 3:**
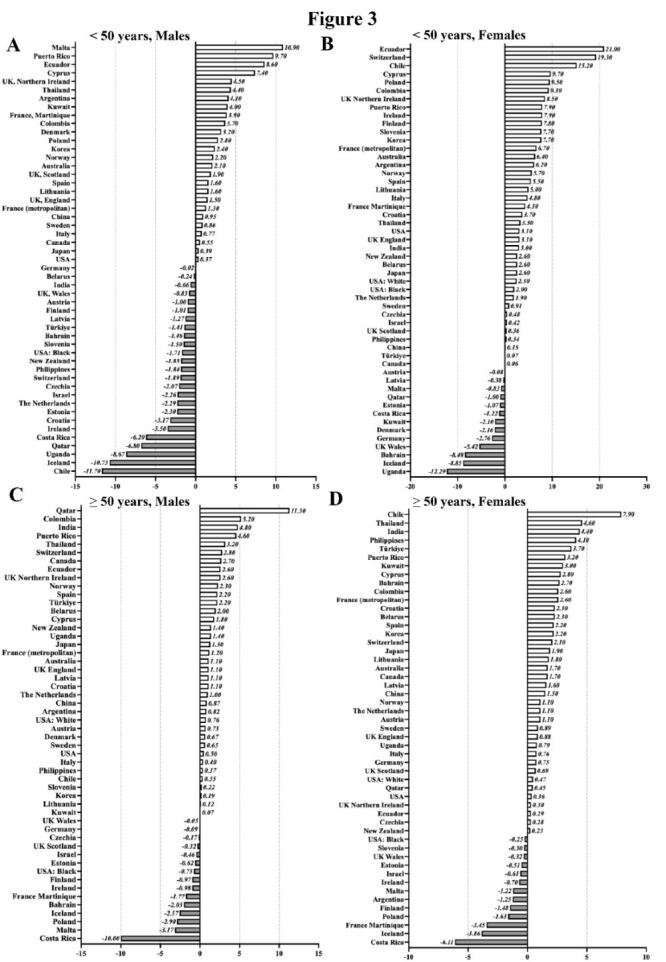
Temporal trends in PC incidence across age and sex groups. EAPC in PC incidence stratified by age and gender into four groups. **Panel A. **< 50 years, males. **Panel B. **< 50 years, females. **Panel C. **≥ 50 years, males. **Panel D. **≥ 50 years, females. [18]. EAPC – estimated annual percentage change, PC – pancreatic cancer.

We analysed the EAPCs in incidence for PC across 52 countries/regions, stratified by gender (male and female) and age (<50 and ≥ 50 years) to evaluate recent global trends (**Figure 3**).

Within the younger population (age < 50 years), incidence trends varied considerably. Among males, PC incidence ascended in n/N = 26/52 countries/regions, with Malta having the highest increase (EAPC = 10.90) and largest decrease in Chile (EAPC = −11.70) (**Figure 3**, panel A), respectively. In contrast, among females, incidence rose in n/N = 39/52 countries/regions, with Ecuador showing the highest value (EAPC = 21.00) and greatest decrease in Uganda (EAPC = −12.29) (**Figure 3**, panel B).

In the elder population (≥ 50 years), PC incidence increased in most countries, rising in n/N = 37/52 countries for males (notably Qatar, EAPC = 11.30) and in n/N = 38/52 for females (led by Chile, EAPC = 7.90). While Costa Rica had the sharpest declines in both males (EAPC = −10.00) and females (EAPC = −6.11) (**Figure 3**, panels C–D).

Overall, these findings reveal a consistent growth in PC incidence across all age and gender groups, especially among the elderly group and younger females. This pattern suggests a growing concern for younger females and also indicates a significantly increasing global burden of PC in ageing populations worldwide.

### Trends in mortality stratified by age and gender

To assess recent trends of global PC mortality, we analysed the EAPCs across 68 countries/regions, stratified by gender (male and female) and age (< 50 and ≥ 50 years (Figure S1 in the **Online Supplementary Document**).

Among younger groups (< 50 years), PC mortality generally declined. Increases occurred in 24 countries/regions for males with the notable exceptions in Mauritius (EAPC = 10.50); and 28 for females with the highest in Uzbekistan (EAPC = 12.90). The most pronounced decreases were observed in Belize (EAPC = −18.73) for males and Kuwait for females (EAPC = −18.79; Figure S1, Panels A–B in the **Online Supplementary Document**).

Among males aged ≥ 50 years, PC mortality increased in n/N = 29/68 countries with the highest increase in Georgia (EAPC = 7.60); one country showed no change, and mortality decreased in 38 countries, with the greatest decline in Panama (EAPC = −4.15) (Figure S1, panel C in the **Online Supplementary Document**). Nonetheless, in sharp contrast, for females aged ≥ 50 years, PC mortality increased in 45 countries/regions and decreased in 23 countries/regions, with the highest EAPC in Georgia (EAPC = 6.80) and the lowest in Belize (EAPC = −6.55) (Figure S1, panel D in the **Online Supplementary Document**).

Taken together, the most noteworthy finding is the concerning increase in PC mortality among older females. This trend distinguishes them from other demographic group and highlights an urgent public health priority requiring targeted interventions for this population.

### Projections of PC burden from 2020 to 2050

To project future trends in PC incidence and mortality, we estimated the number of new cases and deaths (in thousands) from 2022 to 2050, stratified by age (< 50 years and ≥ 50 years), gender (male and female), and continent (Figure S2 in the **Online Supplementary Document**).

Globally, the burden of PC is slightly higher in males than in females, with a clear age divergence in projected trends. By 2050, the elderly (≥ 50 years) will account for the majority of new cases and deaths. Among this group, new cases are projected to rise by 85.1% in males (n = 458 903) and 87.7% in females (n = 430 112); while deaths will increase by 86.7% (males = 437 235) and 91.5% (females = 405 566). In contrast, the younger populations (< 50 years) will see minimal growth, with new cases rising by 17.8% in male and 17.1% in females, and death rising by 19.2% and 20.7%, respectively (Figure S2, panels A–B in the **Online Supplementary Document**).

Across continents, Asia will face the heaviest absolute burden, with 487 087 estimated new cases and 458 647 deaths by 2050, while Africa is expected to experience the fastest growth: incidence is projected to rise by 156.7% and mortality by 159.2%, far exceeding growth rates in other continents (Figure S2, panels C–D in the **Online Supplementary Document**).

Overall, the statistics predict a drastic rising trend in PC incidence and mortality from 2022 to 2050, with a heavy burden in ageing populations. Significant regional disparities are anticipated, with emerging crisis in Africa (fastest growth) and Asia (largest absolute burden).

### HDI and PC in 2022

Next, stratification by HDI revealed consistently higher incidence and mortality rates of PC in countries with higher HDI levels (Figure S3, panel A in the **Online Supplementary Document**). However, the MIR was lowest in very high HDI nations and increased progressively as HDI decreased (Figure S3, panel B in the **Online Supplementary Document**). As for 5-year prevalence, Uruguay demonstrated the highest rate (11.21/100 000), followed by Hungary (10.49/100 000) and France (9.74/100 000). The lowest prevalence was observed in Vanuatu (0.55/100 000), Malawi (0.74/100 000), and Pakistan (0.78/100 000) (**Table 1**; Figure S3, panel C in the **Online Supplementary Document**). As anticipated, PC exhibited positive correlations with national HDI levels (*r* = 0.755, *P* < 0.001) (Figure S3, panel C in the **Online Supplementary Document**). The full picture of PC epidemiological profiles is presented in **Table 1**.

### Lifestyle-associated risk factors and PC

The Spearman’s correlation analysis revealed significant associations between lifestyle-associated risk factors and PC prevalence across sex groups. Strong positive correlations were observed for raised total cholesterol (*r* = 0.695, *P* < 0.001), cigarette smoking (*r* = 0.528, *P* < 0.001), alcohol drinking (*r* = 0.505, *P* < 0.001), obesity (*r* = 0.376, *P* < 0.001), and insufficient physical activity (*r* = 0.204, *P* = 0.006) with both sexes in PC prevalence (Figure S4, panel A in the **Online Supplementary Document**). In males, hypertension was an additional significant risk factor (*r* = 0.382, *P* < 0.001) (Figure S4, panel B in the **Online Supplementary Document**). In females, obesity and insufficient physical activity showed no significance, whereas hypertension showed a rare negative correlation (*r* = −0.259, *P* < 0.001) (Figure S4, panel C in the **Online Supplementary Document**) – a potential outlier warranting further investigation.

In logistic regression analysis, countries were divided into two groups based on the median PC prevalence cutoffs of 3.88/100 000 for both sexes, 4.25/100 000 for males and 3.52/100 000 for females. Three factors emerged as independently significant for both sexes: raised total cholesterol (OR = 1.059; 95% CI = 1.012–1.107, *P* = 0.012), cigarette smoking (OR = 1.136; 95% CI = 1.063–1.214, *P* < 0.001), and alcohol drinking (OR = 1.094; 95% CI = 1.047–1.144, *P* < 0.001); the combined predictive model demonstrated an exceptional discrimination (AUC = 0.908, *P* < 0.001) (Figure S5, panel A in the **Online Supplementary Document**). Among males, hypertension (OR = 1.085; 95% CI = 1.015–1.160, *P* = 0.016), raised total cholesterol (OR = 1.051; 95% CI = 1.011–1.160, *P* = 0.012), cigarette smoking (OR = 1.059; 95% CI = 1.018–1.102, *P* = 0.004), and alcohol drinking (OR = 1.057; 95% CI = 1.024–1.092, *P* = 0.001) were of significance; with a good predictive model to identify high-prevalence countries, AUC = 0.897, *P* < 0.001 (Figure S5, panel B in the **Online Supplementary Document**). In females, significance was found in raised total cholesterol (OR = 1.075; 95% CI = 1.022–1.131, *P* = 0.005), cigarette smoking (OR = 1.167; 95% CI = 1.072–1.271, *P* < 0.001), and alcohol drinking (OR = 1.105; 95% CI = 1.039–1.176, *P* = 0.002); with a good predictive model, AUC = 0.902, *P* < 0.001 (Figure S5, panel C in the **Online Supplementary Document**).

## DISCUSSION

This study delivers a systematic examination of worldwide PC epidemiological trends, uncovering substantial variations in disease prevalence among different geographical areas, age, and gender categories. Geographically, the most pronounced PC burden lies in two continents, with the highest incidence and mortality showing in Northern America and Europe, respectively. Socioeconomic analysis revealed countries with higher HDI accounted for a greater burden of PC, whereas low-HDI countries consistently demonstrated higher MIRs. This epidemiological pattern primarily stems from two key factors: 

(1) western lifestyles and the condition/disease brought about (*e.g*. diabetes mellitus, obesity, lower HDL cholesterol levels, *etc*.) [25,26], and 

(2) the regional disparities on health care system capacities (*e.g*. access to effective treatment) [27].

Moreover, Asia is anticipated to bear the highest burden (487 087 cases by 2050), reflecting its large ageing demographic and rising prevalence of metabolic disorders such as diabetes [28]. Conversely, Oceania’s low incidence may correlate with its small population and earlier adoption of preventive measures. Africa's incidence and mortality growth rates are the highest of any continent, underscoring a critical lack of effective policies and investments in prevention, early detection, and treatment services [29].

Consistent with previous findings [30–32], males showed higher PC incidence and mortality than females. This sex-based disparity may involve several mechanisms: first, androgens in males exert immunosuppressive effects, while oestrogens in females increase susceptibility to autoimmune disorders [33–35]; second, males are more prone to high-risk behaviours such as smoking and alcohol use, amplifying genetic and hormonal risks [36–38]. These factors collectively disrupt metabolic, immune, and inflammatory pathways, ultimately impairing genetic fidelity [33,39]. Notably, recent trends and 2050 projections indicate a faster rise in female incidence, potentially linked to increased consumption of red/processed meats [40,41], sugar-sweetened beverages [42–44], and refined carbohydrates, and rising obesity rates [45–47].

Existing evidence have proved that ageing is a prevailing contributor to carcinoma [25,48]. As populations age globally, cancer risk increases. Accordingly, most PC cases are diagnosed in the elderly. Future projections also show a rising PC burden, largely driven by ageing. However, current PC incidence is increasing in young people, particularly young females. Possible reasons include family history or germline mutations [49,50], cigarette smoking [51], excess body weight [52] and sexual precocity with marked symbols like an early onset of menarche [53].

In addition, our findings corroborate and extend existing evidence on the pivotal role of modifiable lifestyle factors in pancreatic carcinogenesis. Our analyses consistently identified key risk factors: smoking [51], alcohol drinking [54], obesity [52], and raised total cholesterol [55]. Metabolic dysregulation can be rendered as a central driver. Raised cholesterol was strongly linked to PC prevalence, potentially by promoting inflammation [56] and insulin resistance [57]. Notably, the gender-stratified analysis revealed hypertension was a male-specific risk, possibly reflecting sex differences in androgen-mediated metabolic pathways [58]. The negative correlation between hypertension and PC in females warrants further investigation, potential protective effects of antihypertensive medications (*e.g*. ACE inhibitors) can be hypothesised [59–62]. Gender-tailored strategies may yield greater benefits for both sexes.

While this study leverages robust methodologies, with multinational registries (GCO/GHO) and population-wide sampling, its reliance on macro-level data sets inherently obscures subnational epidemiological heterogeneities. Temporal constraints further limit the detection of emergent trends, particularly in evolving risk factors like hormone-mediated carcinogenesis. Augmenting current findings with longitudinal individual-level data, stratified by biologic sex and geographic granularity, might advance precision in PC risk modelling [26,63,64].

## CONCLUSIONS

This research identifies the global epidemiological profiles, modifiable risk factors and future projections of PC. Northern America and Europe currently bear the highest burden. Projections imply upcoming challenges for Asia with the largest estimated cases and Africa with the greatest accelerating rate, making swift intervention an urgency. Remarkable age and gender-related divergence exist: males face higher PC risk than females, and incidence and mortality rise sharply with age. An alarming increase in incidence among young females also warrants attention. Key modifiable risk factors include raised total cholesterol, obesity, alcohol use, and smoking.

To sum up, our data highlight the need for region-specific and population-tailored preventive strategies, particularly in high-burden regions such as Northern America and Europe, as well as in Asia and Africa where future risks are elevated. Subsequent researches should leverage longitudinal individual-level data, disaggregated by sex and geography, to better understand contemporary PC epidemiology and formulate concrete responses to emerging challenges.

## Additional material


Online Supplementary Document
